# FACES OF FORTITUDE: DIVERSE CAREGIVERS PROVIDING COMPLEX CARE

**DOI:** 10.1093/geroni/igae098.0770

**Published:** 2024-12-31

**Authors:** Heather Young, Janice Bell, Kathleen Kelly

**Affiliations:** Chair; Co-Chair; Discussant

## Abstract

Last year, more than 56 million family caregivers provided unpaid care to a family member, a number that will grow along population aging and increased prevalence of chronic and serious illness. The demands, resources and outcomes of family caregiving are shaped by the social and environmental context and the particular demands associated with the condition of the care recipient. In 2021, California adopted a Master Plan on Aging, including two investments to strengthen community-based supports for caregivers: Improving caregiver resource centers and adult day programs. This mixed method symposium highlights the fortitude of family caregivers and community-based organizations. We focus on specific health conditions in understudied family caregiver populations as well as the interface with formal services. The first paper examines experiences of caregiving and home care among caregivers of persons with Amyotrophic Lateral Sclerosis. The second paper compares caregiving activities and outcomes among caregivers of individuals with Parkinson’s Disease/Lewy Body Dementia to those with Alzheimer’s Disease and related dementia and other chronic conditions. The third paper examines building family capacity through family and nurse collaboration during acute illness. The fourth presentation describes the impact of adult day care services from the perspectives of family caregivers of persons with dementia. The final presentation, using a large statewide database, describes caregiving demands, unmet needs and resource gaps of diverse family caregivers to inform a caregiving equity plan for the State of California. Together, these papers reflect complexity, diversity and fortitude in family caregiving.

